# Interprofessional Needs Analysis and User-Centred Prototype Evaluation as a Foundation for Building Individualized Digital Education in Dementia Healthcare Supported by Artificial Intelligence: A Study Protocol

**DOI:** 10.3390/healthcare11101508

**Published:** 2023-05-22

**Authors:** Manuela Malek, Julia Nitsche, Claudia Dinand, Jan Ehlers, Vanessa Lissek, Patricia Böhm, Eva-Maria Derksen, Margareta Halek

**Affiliations:** 1 School of Nursing Science, Witten/Herdecke University, Alfred-Herrhausen-Straße 50, 58455 Witten, Germany; 2 School of Didactics and Educational Research in Health Care, Witten/Herdecke University, Alfred-Herrhausen-Straße 50, 58455 Witten, Germany; 3 School of Psychology, Ruhr-Universität Bochum, Universitätsstraße 150, 44801 Bochum, Germany; 4 Fachhochschule Münster, Corrensstraße 25, 48149 Münster, Germany; 5 Alfried Krupp Krankenhaus Rüttenscheid, Alfried-Krupp-Straße 21, 45131 Essen, Germany

**Keywords:** digital education, individual education, learning preferences, artificial intelligence, dementia, behavioural change

## Abstract

Continuous profession-specific training is necessary to provide high-quality care for people with dementia. Research shows the need for more educational programmes that are personalized and responsive to the learning needs and preferences of staff. Digital solutions supported by artificial intelligence (AI) may be a means of making these improvements. There is a lack of formats that support learners in selecting the right content according to their learning needs and preferences. The project “My INdividual Digital EDucation.RUHR” (MINDED.RUHR) addresses this problem and seeks to develop an automatized delivery system for individual learning content using AI. The sub-project presented here aims to achieve the following: (a) explore learning needs and preferences regarding behavioural changes in people with dementia, (b) develop learning nuggets, (c) evaluate the feasibility of the digital learning platform, and (d) identify optimization criteria. Following the first phase of the framework for the design and evaluation of digital health interventions (DEDHI), we use a qualitative approach with focus group interviews for exploration and development, and co-design workshops and expert audits to evaluate the developed learning nuggets. The developed e-learning tool is the first step in supporting the digital training of healthcare professionals in the context of caring for people with dementia, individualized through AI.

## 1. Introduction

In Germany, 1.7 million people are living with dementia. Two-thirds of them are 80 years old or older, and approximately 20,000 are younger than 65 years old. Provided that there are no significant changes in prevention and treatment, the number of people living with dementia will increase by 40,000 every year and rise to approximately 3 million by 2050 [1]. This disease is a nonspecific clinical syndrome resulting from a chronic and irreversible neurocognitive disorder, with persistent effects on daily living skills with behavioural changes over time. In addition to the well-known Alzheimer’s dementia, there are several less common and heterogeneous clinical syndromes with different clinical symptoms [2]. Specific knowledge and different therapeutic, nursing, and psychosocial interventions are needed for different forms of dementia in order to provide high-quality care. In the course of living with the disease, people with dementia are cared for by different healthcare professionals. In order to provide high-quality care, profession-specific and transdisciplinary knowledge is needed for each dementia type, stage, manifestation, care arrangement, and requirement. Constantly actualized knowledge must be integrated into the daily work routine [3]. The German National Dementia Strategy calls for specific dementia-sensitive care concepts, qualification requirements, and a multiprofessional perspective. Person-centred and interprofessional teamwork is important for best-care practice. It is necessary that all staff members who work in dementia-related healthcare receive a basic qualification in dementia care. The contents of this qualification are to be complementary and additive, as they are not included in general education [4].

Behavioural change in people with dementia is a particular challenge for the people who surround them. Such behavioural and psychological symptoms include agitation, depression, apathy, repetitive questioning, psychosis, aggression, sleep problems, and wandering. Managing these symptoms is among the most problematic and distressing aspects of care. This behaviour can be caused by a loss of abilities as well as by an inadequate environment [5,6]. The actions of staff, particularly their recognition of trigger situations, can play a central role in modifying challenging behaviours in people living with dementia [7]. It can be helpful for healthcare professionals to learn to understand the different messages in their patients’ actions so that they can recognize that many challenging behaviours have particular meanings. Studies also confirm that staff working in dementia care require information on how to respond to emergent behaviours, the use of correct body language, and how to deescalate behaviour. More needs analysis is required to define profession-specific knowledge requirements [8]. Approaches that are adapted to the patient and caregiver are also needed. This will provide individuals with the chance to assess behaviours and their contexts, enabling them to act in an adequate and person-centred manner [9,10]. The literature provides a great deal of information that can be used across health professions to manage challenging behaviours in a professional and understanding way [11,12]. For example, communication is key to an effective intervention, enabling carers to understand the challenging needs and wishes of people living with dementia [13]. However, successful implementation requires widespread expertise, which should also be provided in a user-oriented format. However, the existing learning formats are often inflexible for heterogeneous user groups and individual learning preferences. The integration of individual learning preferences is a key factor that prevents users’ frustration at or rejection of the format. It is already possible to produce specific content for each learning preference using the current e-learning development technologies. However, there is a lack of actionable solutions to support the learner in selecting the right content and to offer and recognize preference-specific content [14].

In recent years, e-learning recommender systems have become increasingly popular because, unlike learning management systems, they use intelligent algorithms to enable adaptive individual adjustment to learning types and learning performance, thereby also influencing learner satisfaction [15]. Traditional learning management systems are currently losing users. This may be because learners cannot interact with the platform and there is no individual adaptation [16]. However, in order to enable this interaction and individualization, an environment is needed that addresses the learning preferences and performance of the learner [17].

A special focus on learning preferences is adopted because each learner has their own learning style, to which the content and methods should be adapted [18]. Thus, each learning style also provides information for the development of the learning content, which should be used accordingly in the design in order to generate the highest possible learning success [19]. The current technical possibilities now make it feasible to respond to the needs of learners in a much more individualised way and, thus, to increase learning success and make the system more efficient in the medium term.

Various types of learning models can be found in the literature. In the context of adaptive learning systems, the Felder–Silverman learning style model is one of the most widely used and thus most widely adapted [20]. It provides a combination of advantages from other learning style models, such as Kolb’s learning style model [21], Pask’s theory [22], or even the Myers–Briggs Type Indicator [23]. The Felder–Silverman learning style model consists of four dimensions in which learners’ learning styles (two per dimension) can be classified [24]. With this, as well as with other learning style models, a sharp separation of the individual types is not always possible; therefore, in application research, it must be decided which model is the most suitable.

In healthcare, digital media are increasingly supplementing education and training. During the COVID-19 pandemic, training on digital media was used more in healthcare than in any other industry. Out of necessity, many clinics and academics have already developed their own individual solutions. However, these individual solutions offer minimal possibilities for interoperability. This usually results in separate learning worlds without the possibility of exchange. Practical solutions are needed [25]. 

Artificial intelligence (AI) is becoming increasingly important in the education sector and is an important tool for personalized and individual learning settings. It can also be applied to adaptive learning systems. AI systems can support learners individually and increase their learning progress, addressing learners’ specific learning needs and preferences. For example, AI offers the possibility of creating individual learning paths for learners. This adaptation to individual needs improves learning situations, experience, and acceptance [26,27]. Adaptive learning systems using the Yixue Squirrel AI learning system were shown to produce greater learning gains than classroom instruction by human educators [28]. Personalized training has a positive impact on learning success [29,30]. Furthermore, AI has been used to create intelligent tutoring systems, extending their adaptive and reactive capabilities for the development of individualized learning environments [31]. Based on current systematic mapping, it is recommended that further research should use adaptive approaches for AI-enabled learning systems. This may increase the use of AI-enabled learning systems and address the challenges faced by learners. From the literature mapping, it is clear that most AI-enhanced learning interventions are made during the experimental phase, and hardly any AI-enhanced educational programs are used in educational practice. Therefore, when developing AI-enhanced learning systems, it is important that developers consider aspects that contribute to the increased use of such systems in practice [32].

**Figure 1:**
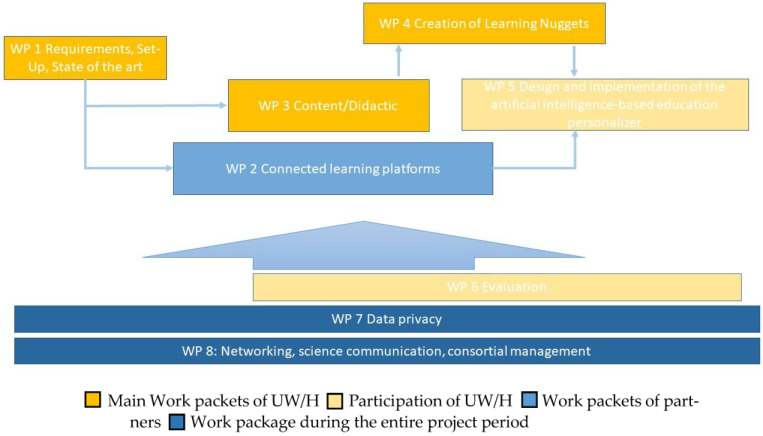
Flowsheet of MINDED.RUHR and relevant work packages for the subproject MR_UW/H.

The project “My INdividual Digital EDucation.RUHR” (MINDED.RUHR) is a collaborative, interdisciplinary project that aims to develop, implement, and evaluate a digital intervention, here, an adaptive learning platform for healthcare professionals and other personnel in dementia care. The digital learning platform should provide individualized educational content for different healthcare professionals in the German healthcare sector and for different learning types. MINDED.RUHR is funded by the Federal Ministry of Education and Research (BMBF) and operationally supported by the Federal Institute for Vocational Training (BIBB) with eight partners (see Figure 1). The included subproject MR_UWH “Development and preparation of individual learning content using the example of challenging behaviour of people living with dementia” is the scope of this study protocol. It aims to explore learning needs, as well as develop, prepare, and evaluate adaptive learning content, which will result in a digital intervention in the form of learning nuggets. MR_UWH will be conducted in an interdisciplinary manner by researchers of didactics and educational research in healthcare and nursing science. 

## 2. Methods

### 2.1. Study Design

A qualitative study design using focus groups, co-design workshops, and an expert audit was chosen based on the DEDHI framework for the design and evaluation of digital health interventions. Within this framework, digital health interventions (DHIs) are defined as “tools and services that use information and communication technologies (ICTs) to improve the prevention, diagnosis, treatment, monitoring, and management of health and lifestyle” [33,34]. In the presented project, the developed learning nuggets form the intervention. DEDHI supports researchers and practitioners during the process of designing and evaluating digital health interventions, and during their implementation. It is structured into three phases [33]. This project focuses on the first phase (the preparation phase), which is divided into the following four steps (see Table 1):

(a) Review the existing justificatory knowledge for the development of a digital health intervention (DHI);
(b) Develop a conceptual model of the DHI outlining the outcomes and intervention components;
(c) Conduct a feasibility and acceptability study to test novel DHI components;
(d) Identify an optimization criterion that provides the best expected outcome within technical and health-related economic constraints.

### 2.2. Participants

The participants involved in the focus groups and co-design workshops are nurses (inpatient and home care), medical professionals, and nursing and medical students, as well as medical assistants, healthcare volunteers, facility managers, and physiotherapists.

Purposive sampling will be used to recruit between 48 and 64 participants for 8 focus groups. The sample size is based on recruiting six to eight participants per group, which is the current recommendation for focus groups [35]. Ideally, for the co-design workshop, the participants will be the same as for the focus groups.

For the recruitment of the entire survey (focus groups and co-design workshops), the project team defined specific criteria.

The inclusion criteria are as follows:

1. Healthcare profession or undertakes daily work in secondary processes;
2. Contact with people with dementia at work.

The exclusion criteria are as follows:

1. No contact with people with dementia at work.

The participants will be assigned to a focus group and co-design workshop based on their profession. According to the inclusion and exclusion criteria, two consortia partners will recruit interview partners. The recruitment process will be conceptualized during regular meetings with the involved partners before data collection to ensure a consistent approach and fit. Staff from both institutions will be invited to participate either via a recruitment email or in person, being provided with all the relevant information and an invitation to contact the research team for further information. The recruitment form will include participant information and consent. Recruitment challenges are anticipated because most health professionals undertake shift work and could be experiencing staff shortages due to the pandemic. Fieldwork pragmatics (e.g., timelines of participants and access to potential participants) will limit the project’s success.

For the audit described in step (c), a maximum of eight experts will be identified. Experts are defined as having specific knowledge and experience of a particular issue. Process knowledge about education organization (e.g., action processes, interaction routines, organizational constellations, and past or current events) is particularly important. The expert is employed in a healthcare organization or works very closely with the daily business and has detailed knowledge of it [36]. For the needs assessment, nursing staff (e.g., a nursing expert for dementia), medics, and people in service and management positions (e.g., residential area management, management of dementia consulting, case management, medical services, representation of interests of the German Alzheimer Society, and social workers) will be selected. Expertise refers to professional knowledge in dealing with behavioural changes in people with dementia based on care, competences, processes, and structures in the healthcare system. Interviews with experts in (digital) didactics are conducted for the development of digital education using AI.

### 2.3. Data Collection

For better understanding and traceability, data collection is structured *according to* the steps of the DEDHI framework.

(a) Review and exploration

For data collection, profession-specific focus group interviews will be conducted using a semi-structured interview guide. Additionally, the usual sociodemographic data of the participants (gender, age, education, profession, and work experience) will be collected digitally using Lime Survey (Version 3.28.6).

The interview guide will be developed by the authors and has two content-related parts. The first part will cover knowledge regarding behavioural changes in people with dementia, including perceptions and impressions of people with dementia, problems and challenges, communication and relationships, competences, and general key knowledge. The second part of the interview includes questions about specific learning preferences, individual learning needs, factors that facilitate or hinder learning implementation, and learning flows. Questions can be prioritized, skipped, or elaborated on to keep the interview flowing.

(b) Development

The results of the interviews are the basis for the development of the learning content and didactics. Finally, a learning preference matrix can be created to assign personnel. According to the identified knowledge needs and relevant topics, recent literature will be used to develop theory- and evidence-based learning content. The literature search will include evidence-based guidelines, recommendations, reviews, and other relevant key information. These results will provide a foundation for technical project partners to create a digital education programme individualized by AI. Addressing research step (b), the research team is supervising the development process of a prototype created by the technical consortia partners.

(c) and (d) Conduct, evaluation, and optimization

The third step consists of co-design workshops addressing research step (c). It will be very important to provide a sense of collaborative work by allowing participants time to personally gather, analyse, and reflect on the relevant feedback. The aim is to explore how participants handle the prototype. The participants in the co-design workshops will work through the prototypical learning nuggets with test accounts. The first step will involve intuitive processing, in which the participants will behave as if they were using the learning nugget in a real situation. Subsequently, content-related and didactic questions will be asked that focus on the experiences of the participants. While the participants are performing and answering the questions related to the various tasks, their information is audio recorded. A cooperative evaluation method is chosen; therefore, there is an active dialogue between the evaluator and the volunteer [37]. Feedback is relevant in terms of the technical and didactic aspects and content design when optimizing the product (d). Two project members monitor and facilitate the activities. Subsequently, the developed learning content will be approved by all authors and audited by experts. The experts will be recruited from the expert group described above according to the learning topics. Feedback from the experts is obtained using a catalogue of criteria (e.g., preparation of the content, actuality, correctness, usability, and didactic preparation). Potential modifications will be implemented.

### 2.4. General Information

Interviews and workshops will be audio recorded (the planned average recording lengths are 120 min and 60 min, respectively). We plan to conduct the interviews on location. If the current pandemic regulations do not allow for this, the interviews will be conducted virtually. Participants will provide written informed consent before data collection. Data collection will be conducted by researchers with experience in dementia care and research and a background in nursing science, and by researchers with experience and expertise in didactics and educational research in healthcare.

Data collection will be completed by March 2022 and that data analysis will be completed by July 2022. The start of the evaluation process, with preparation, is planned to have taken place by May 2023. Therefore, the co-design workshops are planned for March 2023.

### 2.5. Data Analysis

(a) Review and exploration

The recorded interviews will be professionally transcribed verbatim and loaded into MAXQDA 2020 software for data management and analysis. This software was chosen to manage the text data and to organize the codes that were assigned to the transcripts. The data will be analysed thematically and in terms of content, using the Mayring technique [38]. The main categories are to be defined deductively by relying on the interview guide, and the subcategories will be added inductively. First, the project team members of this work will carefully read the transcripts, and their initial thoughts will be documented. Open coding will be conducted by project staff, in which sentences with significant meanings will be assigned initial conceptual labels. The academic research team will collectively discuss these initial codes to ensure intersubjectivity [39].

(b) Development

According to the topics identified in step a) (review and exploration), content for the learning nuggets will be extracted from the identified literature using databases, e.g., Medline (PubMed) and CINAHL. In regular meetings, the project teams will discuss and modify the digitally processed learning nuggets.

(c) and (d) Conduct, evaluation, and optimization

Recorded responses and feedback from the co-design workshops will be professionally transcribed according to simple notation and paraphrasing. Data management and analysis are the same as in the “review and exploration” step. The results will be used to revise the prototypes and the didactic and content recommendations. Team members will document feedback and important comments from expert audits regarding the produced learning nuggets (focusing on learning content and didactic preparation). Important documented feedback will be included and therefore adapted in the learning content.

## 3. Discussion and Limitations

Within the ageing population, the number of people with dementia is expected to increase; with this comes the need for high-quality advanced dementia education for healthcare workers. It is imperative that care workers have the skills and knowledge to provide high-quality, easy-access dementia care. In order to achieve a good level of education for health professionals working in dementia care, thus facilitating person-centred care, we recommend the development of educational programs related to the behavioural and psychological symptoms of dementia that are responsive to staff learning styles [8].

It is important to determine the appropriate content for the learning modules. These are presented in a prioritized learning preference matrix. In order to create learning nuggets for the prototype, storyboards are passed on to the e-learning editorial team, which uses them to create various media (e.g., text, videos, podcasts, and simulations). The AI can then assign the various learning media individually to the learners, depending on their preferences. The technology of AI is highly related to adaptive learning systems. It refers to technologies that dynamically adjust to the level of the individual’s abilities and their general knowledge. This learning system enables the development of individual learning programs and supports learner motivation. As a result, learners’ potential and success are maximized [27]. Overall, the use of AI-based personalized learning settings can offer a valuable opportunity to improve learners’ performance and address individual needs and interests. Study results confirm that individualized and personalized training has a positive impact on learning success. However, it is difficult to organize and provide one-to-one support for each person. Therefore, AI has the potential to be used for adaptive learning environments [29,30].

In order to assess learner preferences, personae are being developed based on the results of this research. The literature has shown that the development of personae helps to create learning modules that an AI can use to suggest data-driven individualized learning pathways [40]. In order to enable the true individualization of learning and not fall into stereotypes [41], this project goes beyond simply using personae through the subsequent use of AI. The personae used here serve primarily for the initial design of the learning nuggets, and the suggestion of individual learning paths is subsequently taken over by the AI.

The results of the qualitative investigation are presented as personae in terms of learning experiences, expectations, job profiles, media competence, obstacles, and so on. These are used to support the AI in the decision-making processes.

The aim of this study is to provide content for digital, individualized, transdisciplinary education for healthcare professionals and other people working in healthcare systems. Based on the data collection, personae are created, and dementia-specific content is developed. Creating a persona, dementia-specific content, and a learning preference matrix is necessary to provide the AI with a basis for the learning process.

Therefore, it is important to show the long-term impact of the project. As already described, the results of the data collection (Figure 1: work packages 3 and 6) will provide a foundation for technical consortia partners to create a digital education programme that is individualized by AI for healthcare professionals who work with people living with dementia in daily practice (Figure 1: work packages 4 and 5). When digital individualized education systems are created, they will be made available to healthcare systems via a digital learning platform. Designing a framework for long-term education may constitute an important step in ensuring the continued implementation of high-quality dementia care.

One challenge concerns the individualization of the content and learning methods or level of depth of the content. The team must recognize the limits and specify the content to a level that can be achieved.

The recruitment process has strengths and limitations. The partners have a personal professional connection to the participants. Human resources development or management staff of the organizations know contactable persons and their staff and processes, such as the service schedule. It is assumed that the recruitment of participants will be easy and uncomplicated; this could be critical, because workers may feel obliged to participate. Because of this, the partners address voluntariness and willingness in a consented procedure.

In the interviews, the project staff expected to encounter negative comments about and rejections of digital training and the current framework. Openness and increasing motivation regarding digital learning could be achieved with the use of gamification elements, such as points, challenges, or badges [42]. However, this is a chance to involve participants in a user-centred process and ask for solutions and ideas for good e-learning. 

## Figures and Tables

**Table 1:** Presentation of the applied steps of DEDHI (Phase 1) applied by Kowatsch [33].

	Approach	Description	Focus Groups	Expert Audit	Co Design Workshops	Others
Steps of DEDHI (Phase 1)						
(a) review existing justificatory knowledge for the development of a DHI		Review and explore the knowledge of needs of health care professionals for caring for people with dementia in different settings and individual and profession-specific learning preferences	eight are planned with health care professionals			literature review
(b) develop a conceptual model of the DHI outlining the outcomes and intervention components		Develop learning nuggets and supervise creation of the prototype				Building personae
(c) conduct a feasibility and acceptability study to test novel DHI components		Conduct, evaluate and optimize feasibility and acceptability of digital training program (ease of use, adherence, personalization, safety, privacy and security)		3-4 audits	eight are planned	
(d) identify an optimization criterion that provides the best expected outcome within technical and health economic constraints		optimize specific criteria			include the results of Co Design Workshops	

## Data Availability

The materials described in this paper pertain to the study protocol only, and there are no raw data reported. The datasets are currently being collected and analysed and can be made available from the corresponding author on reasonable request.
